# Phase I study of safety and immunogenicity of ADU-623, a live-attenuated listeria monocytogenes vaccine (ΔactA/ΔinlB) expressing EGFRVIII and NY-ESO-1, in patients with who grade III/IV astrocytomas

**DOI:** 10.1186/2051-1426-3-S2-P162

**Published:** 2015-11-04

**Authors:** Marka Crittenden, Keith S Bahjat, Rui Li, Pankaj Gore, Chris Fountain, Bill Hanson, Justin Skoble, Peter Lauer, Aimee L Murphy, Thomas Dubensky, Dirk G Brockstedt, Walter Urba

**Affiliations:** 1 Earle A. Chiles Research Institute, Providence Cancer Center; The Oregon Clinic, Portland, OR, USA; 2 Earle A. Chiles Research Institute, Robert W. Franz Cancer Research Center, Providence Cancer Center, Portland, OR, USA; 3 Providence Cancer Center, Portland, OR, USA; 4 Microneurosurgical Consultants, Portland, OR, USA; 5 Aduro Biotech Inc., Berkeley, CA, USA; 6 Earle A. Chiles Research Institute, Portland, OR, USA

## Background

The neo-antigen EGFRvIII is expressed in multiple tumor types, including high-grade astrocytomas. It is associated with a poor prognosis and resistance to conventional therapies such as chemotherapy and radiation that are part of the standard treatment. We propose that immunization with a live-attenuated *Listeria*-based vaccine, ADU-623, expressing EGFRvIII and NY-ESO-1 will elicit robust tumor-specific immune responses capable of killing EGFRvIII and/or NY-ESO-1-expressing tumor cells and improve survival of the patients. In addition, ADU-623 induces a potent innate immune response that can kill transformed cells even in the absence of neo-antigens. We designed a translational vaccine study to evaluate the safety and immunogenicity of this vaccine in patients with high-grade astrocytomas after standard of care therapy or at progression.

## Methods

Patients with a pathologic diagnosis of WHO Grade III/IV astrocytic tumors that have completed standard of care external beam radiation therapy and concurrent temozolomide followed by adjuvant temozolomide or with radiographic evidence of progression following standard of care radiation and chemotherapy treatment, including those who have gone on to a second surgical resection are eligible. Patients are enrolled and assigned consecutively to one of the following ADU-623 dose level cohorts: Cohort 1 3x10^7^ cfu, Cohort 2 3x10^8^ cfu, or Cohort 3 1x10^9^ cfu, each administered IV on Days 0, 21, 42 and 63. Adverse events are monitored throughout the treatment and patients are followed for up to 24 months. Patients are currently accruing to Cohort 3. The primary objective is to determine the maximum tolerated dose and characterize the safety profile of ADU-623 in patients with treated and recurrent WHO Grade III/IV astrocytomas. Secondary objectives include progression free survival, time to progression and overall survival rates in patients vaccinated with ADU-623. Exploratory studies of EGFRvIII-, NY-ESO-1-, vector-specific and innate immune responses will be performed.

## Trial registration

ClinicalTrials.gov identifier NCT01967758.

